# Acute Post-Exercise Myofibrillar Protein Synthesis Is Not Correlated with Resistance Training-Induced Muscle Hypertrophy in Young Men

**DOI:** 10.1371/journal.pone.0089431

**Published:** 2014-02-24

**Authors:** Cameron J. Mitchell, Tyler A. Churchward-Venne, Gianni Parise, Leeann Bellamy, Steven K. Baker, Kenneth Smith, Philip J. Atherton, Stuart M. Phillips

**Affiliations:** 1 Exercise Metabolism Research Group, Department of Kinesiology, McMaster University, Hamilton, Ontario, Canada; 2 Department of Neurology, School of Medicine, McMaster University, Hamilton, Ontario, Canada; 3 Metabolic and Molecular Physiology Research Group, MRC-ARUK Centre of Excellence for Musculoskeletal Ageing Research, School of Graduate Entry Medicine and Health, Derby, United Kingdom; University of East Anglia, United Kingdom

## Abstract

Muscle hypertrophy following resistance training (RT) involves activation of myofibrillar protein synthesis (MPS) to expand the myofibrillar protein pool. The degree of hypertrophy following RT is, however, highly variable and thus we sought to determine the relationship between the acute activation of MPS and RT-induced hypertrophy. We measured MPS and signalling protein activation after the first session of resistance exercise (RE) in untrained men (n = 23) and then examined the relation between MPS with magnetic resonance image determined hypertrophy. To measure MPS, young men (24±1 yr; body mass index  = 26.4±0.9 kg•m^2^) underwent a primed constant infusion of L-[ring-^13^C_6_] phenylalanine to measure MPS at rest, and acutely following their first bout of RE prior to 16 wk of RT. Rates of MPS were increased 235±38% (*P*<0.001) above rest 60–180 min post-exercise and 184±28% (*P* = 0.037) 180–360 min post exercise. Quadriceps volume increased 7.9±1.6% (−1.9–24.7%) (*P*<0.001) after training. There was no correlation between changes in quadriceps muscle volume and acute rates of MPS measured over 1–3 h (r = 0.02), 3–6 h (r = 0.16) or the aggregate 1–6 h post-exercise period (r = 0.10). Hypertrophy after chronic RT was correlated (r = 0.42, *P* = 0.05) with phosphorylation of 4E-BP1^Thr37/46^ at 1 hour post RE. We conclude that acute measures of MPS following an initial exposure to RE in novices are not correlated with muscle hypertrophy following chronic RT.

## Introduction

Skeletal muscle hypertrophy following resistance training (RT) requires the net addition of new myofibrillar proteins; thus, myofibrillar protein synthesis (MPS) must exceed myofibrillar protein breakdown (MPB). Using contraction-induced phosphorylation as a proxy for activation and activity, signalling pathway proteins in the Akt (PKB)-mTOR pathway have been measured in humans and some [1], but not all [2], have reported correlations between the phosphorylation state of certain proteins and hypertrophy. In larger samples, measures of protein phosphorylation in multiple signaling proteins are not related to the highly variable phenotypic hypertrophic response seen with RT [3]; however, we hypothesized that a stronger relationship would exist between MPS and hypertrophy.

Rates of MPS measured in the fed state have been used to evaluate the effect of exercise and nutritional interventions and their potential to induce muscle hypertrophy following RT [4], [5]. Previous work from our lab has shown that responses of MPS measured after resistance exercise with ingestion of milk or soy protein [4] or carbohydrate (Tang et al., 2007) are in alignment with RT-induced muscle hypertrophy during RT program employing similar exercises and post-exercise nutrition in separate groups of subjects [6]. Similarly, we reported that the acute MPS response with: heavy and light-load fatiguing resistance exercise [7], and with differing volumes of resistance exercise [8] were in agreement muscle hypertrophy following 10 wk of RT in a different set of subjects [2]. Taken together, the congruence between acute MPS responses and chronic RT-induced hypertrophy would suggest that measures of acute post-exercise MPS may vary in a similar manner to, and thus be related to, muscle hypertrophy; however, such a possibility has not been tested.

There is a high degree of variability in the hypertrophic response to RT. Typical coefficients of variation of the hypertrophic response measured using muscle fibre size changes in young and old men and women can exceed 100% [2], [3], [6], [9]. There have been attempts to explain this variability in hypertrophy using gene expression [3], [10], satellite cell enumeration [9], measures of the hormonal response to exercise [11], and measurement of cell signaling proteins [1], [12]. To date, however, there are no published studies addressing the relationship between acute measures of MPS and hypertrophy following RT in the same subjects. Both gene expression [3], [10] and satellite cell content [9] have been shown related to hypertrophy in some instances whereas acute post-exercise systemic hormonal responses show no relationship to RT-induced hypertrophy. In humans, protein signaling has only been shown to relate weakly to hypertrophy [13] or in very small sample sizes [1] and is not consistently observed [2], [12]. The purpose of this study was to determine if acute myofibrillar protein synthesis measured acutely in training-naïve subjects after their first bout of resistance exercise with protein consumption was related to muscle hypertrophy following 16 weeks of RT.

## Methods

### Ethics Statement

All participants were informed of the purpose of the study, the experimental procedures involved and all the potential risks involved before obtaining written consent. The protocol and consent form were approved by the Research Ethics Board of Hamilton Health Sciences and McMaster University and complied with all ethical standards for research involving human participants set by the Declaration of Helsinki and by the Canadian Tri-Council statement on ethics in human research (http://www.ethics.gc.ca/eng/policy-politique/initiatives/tcps2-eptc2/Default/).

### Subjects

Twenty-three healthy young men (177±2 cm; 84.1±3.5 kg; body mass index  = 26.4±1.0 kg•m^−2^; 24±1 yr, means ± SD) participated in the experiment. Subjects were recreationally active but had not engaged in RT within the last year.

### Experimental Design

Participants underwent a magnetic resonance imagining (MRI) scan of their right thigh to determine muscle volume and a dual-energy x-ray absorptiometry (DXA) scan to assess whole body fat- and bone-free mass (lean mass). Subjects were then strength tested to determine their maximal isotonic strength, which is traditionally labelled as one repetition maximum (1RM) for all training exercises. At least 5 d following strength testing participants reported to the lab after a 10 h overnight fast for stable isotope infusion. Resting MPS was measured, subjects then completed four sets of 8 repetitions of leg press, leg extension, leg curl and calf press. They then ingested a protein rich beverage containing 30 g of milk protein, 25.9 g of carbohydrates and 3.4 g of fat (Musahi P30, Notting Hill, Australia). Muscle biopsies were then taken at 1, 3 and 6 hours post exercise to measure MPS. Subjects then completed 16 weeks of RT while ingesting the protein rich beverage immediately after their exercise session and with breakfast on non-training days, as previously described in [12] Briefly, participants trained four times weekly with two upper and two lower body workouts. Lower body exercises are described above in the acute exercise session. Upper body exercises consisted of chest press, shoulder press, seated row, lat pulldown, bicep curl and tricep extension. The program was progressive in linear manner moving from 3 sets of 12 repetitions to 4 sets of 6 repetitions. At the end of the training period, MRI, DXA scans, and strength testing were repeated.

### Infusion Protocol

On the trial day, participants reported to the lab after an overnight fast having refrained from any strenuous physical activity for at least 3 days. A 20-gauge plastic catheter was inserted into an antecubital vein and a baseline blood sample was obtained. Following the start of a primed constant infusion ofL-[*ring-* ^13^C_6_] phenylalanine (prime: 2 μmol kg^−1^; infusion: 0.05 μmol kg^−1^ min^−1^), participants rested for 3 h before a muscle biopsy was obtained to determine the resting (basal) rate of MPS. Subjects then completed the lower body exercise protocol described above and ingested a protein rich beverage (described above). They then rested in bed for the next 6 h while biopsies (*vastus lateralis*) were taken 1, 3 and 6 h after cessation of the exercise bout.

The drink containing 30 g of milk-based protein was enriched to 6% of the protein phenylalanine content with free [^13^C_6_] phenylalanine tracer to minimize disruptions in isotopic steady state, which is an approach we have used numerous times before with good maintenance of isotopic steady-state [14], [15]. Biopsies were obtained with a Bergström needle modified for manual suction under local anaesthesia (2% xylocaine). Biopsy samples were blotted and freed of any visible fat and connective tissue, frozen in liquid nitrogen (within ∼20 s of being taken from the muscle) and stored at −80°C until further analysis.

### Imaging

After arriving at the site of the MRI scanner, subjects rested in the supine position for 1 h prior to scanning to prevent the influence of potential fluid shifts on muscle volume. Subjects were instructed not to engage in any strenuous activity within 24 h of the scanning. MRI scans were performed in a 3-T HD scanner (Signa MRI System; GE Medical, Milwaukee, WI) at the Brain-Body Institute, Imaging Research Centre, St. Joseph's Healthcare (Hamilton, Ontario). Image acquisition was carried out using T1 fluid attenuation inversion recovery in the axial plane with the following parameters: repetition time/echo time  = 2,100 ms/23.8 ms; field of view  = 25–30 cm; matrix size  = 512/512 slice thickness  = 5 mm. Thigh image acquisitions utilized an eight-channel torso coil with two excitations. There was a 10 mm gap between slices. Quadriceps volume was calculated by multiplying the slice area by the distance between slices. Volume was measured from the first slice where the rectus femoris was visible to the first slice where the gluteus maximus was visible. ImageJ software (U. S. National Institutes of Health, Bethesda, MD) was used to determine the area of each slice. Pre- and post-scans were performed at the same time of day and joint angle and leg compression was controlled using a custom built foot frame.

Whole-body DXA scans (QDR-4500A; Hologic, software version 12.31) were carried out pre and post training to determine total body weight, fat mass, and (fat and bone free) lean mass.

### Western Blotting

Muscle samples (∼40–50 mg) were homogenized on ice in buffer (10 *μ*l mg^−1^ 25 mM Tris 0.5% v/v Triton X-100 and protease/phosphatase inhibitor cocktail tablets (Complete Protease Inhibitor Mini-Tabs, Roche, Indianapolis, IN; PhosSTOP, Roche Applied Science, Mannhein, Germany). Samples were then centrifuged at 15,000 *g* for 10 minutes 4°C. The supernatant was removed and protein concentrations were determined via BCA protein assay (Thermo Scientific, Rockford, IL). Working samples of equal concentration were prepared in Laemmli buffer. Equal amounts (20 µg) of protein were loaded onto 10% or gradient precast gels (BIO-RAD Mini-PROTEAN TGX Gels, Bio-Rad Laboratories, Hercules, CA) for separation by electrophoresis. Proteins were then transferred to a polyvinylidene fluoride membrane, blocked (5% skim milk) and incubated overnight at 4°C in primary antibody: phospho-Akt^Ser473^ (1∶1000, Cell Signalling Technology, #4058) phospho-mTOR^Ser2448^ (1∶1000, Cell Signalling Technology, #2971), phospho-4E-BP1^Thr37/46^ (1∶1000, Cell Signalling Technology, #2855), Phospho-S6^Ser240/244^ Ribosomal protein (1∶2000, Cell Signalling Technology, #2215). Membranes were then washed and incubated in secondary antibody (1 h at room temperature) before detection with chemiluminescence (SuperSignalWest Dura Extended Duration Substrate, ThermoScientific, #34075) on a FluorChem SP Imaging system (Alpha Innotech, Santa Clara, CA). Phosphorylation status was expressed relative to α-tubulin (1∶2000, Cell Signalling Technology, #2125). Images were quantified by spot densitometry using ImageJ software (US National Institutes of Health).

### Isotopic Analyses

As described previously [7] approximately 20 mg (wet weight) of muscle was used to isolate free intracellular amino acids. A separate piece of muscle (∼30 mg) was used to isolate, hydrolyse, purify, derivatize and analyse the myofibrillar protein fraction enrichment. The rate of myofibrillar protein synthesis was calculated using the standard precursor–product method as previously described [7]:
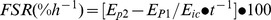



Where, FSR is the fractional synthetic rate, E_p2_ and E_p1_ are the protein bound enrichments from muscle biopsies at time 2 (E_p2_) and plasma proteins or the previous muscle biopsy at time 1 (E_p1_) and thus their difference is the change in bound protein enrichment between two time points; E_ic_ is the mean intracellular phenylalanine enrichment from biopsies at time 2 and time 1; and t is the tracer incorporation time. The utilization of “tracer naïve” subjects allowed us to use the pre-infusion blood sample (i.e., mixed plasma protein fraction) as the baseline enrichment (E_p1_) for the calculation of resting MPS. This approach makes the assumption that the baseline ^13^C enrichment (δ^13^C_PDB_) in the blood reflects that of muscle protein; this is an assumption that has been previously [16] and shown to be valid in allowing calculation of a reliable rate of MPS in the fasted state [17], [18].

### Statistics

Differences in means from pre to post training were compared with parried Student's t-tests. Temporal differences in the phosphorylation of signalling proteins and FSR were compared with one-way repeated measures ANOVA. Relationships between variables were assessed using the Pearson's product moment correlation. All analyses were conducted using SPSS version 20 (IBM Armonk, New York, USA). Alpha was set at *P*≤0.05. Means are reported ± SE. A priori sample size calculations revealed that a sample of 23 subjects would be sufficient to detect a relationship accounting 25% of the variance between gains in muscle volume and increases in MPS with 80% power.

## Results

### Plasma and Muscle Intracellular Free Phenylalanine Enrichment

Intracellular free- phenylalanine precursor enrichments were 0.046±0.003 at rest and 0.066±0.004 throughout the post exercise incorporation period. The slope of a linear regression lines fit through the intracellular enrichments was not significantly different from zero during the post-exercise period (*P*>0.05). Plasma enrichments at 60, 180 and 360 min were 0.070±0.002, 0.075±0.003 and 0.076±0.003, respectively. Linear regression analysis indicated that the slopes of the plasma enrichments were not significantly different from zero (*P*>0.05) and thus an isotopic plateau was achieved and that the use of the steady-state precursor product equation was appropriate.

### Muscle Size and Strength

**Figure 1:**
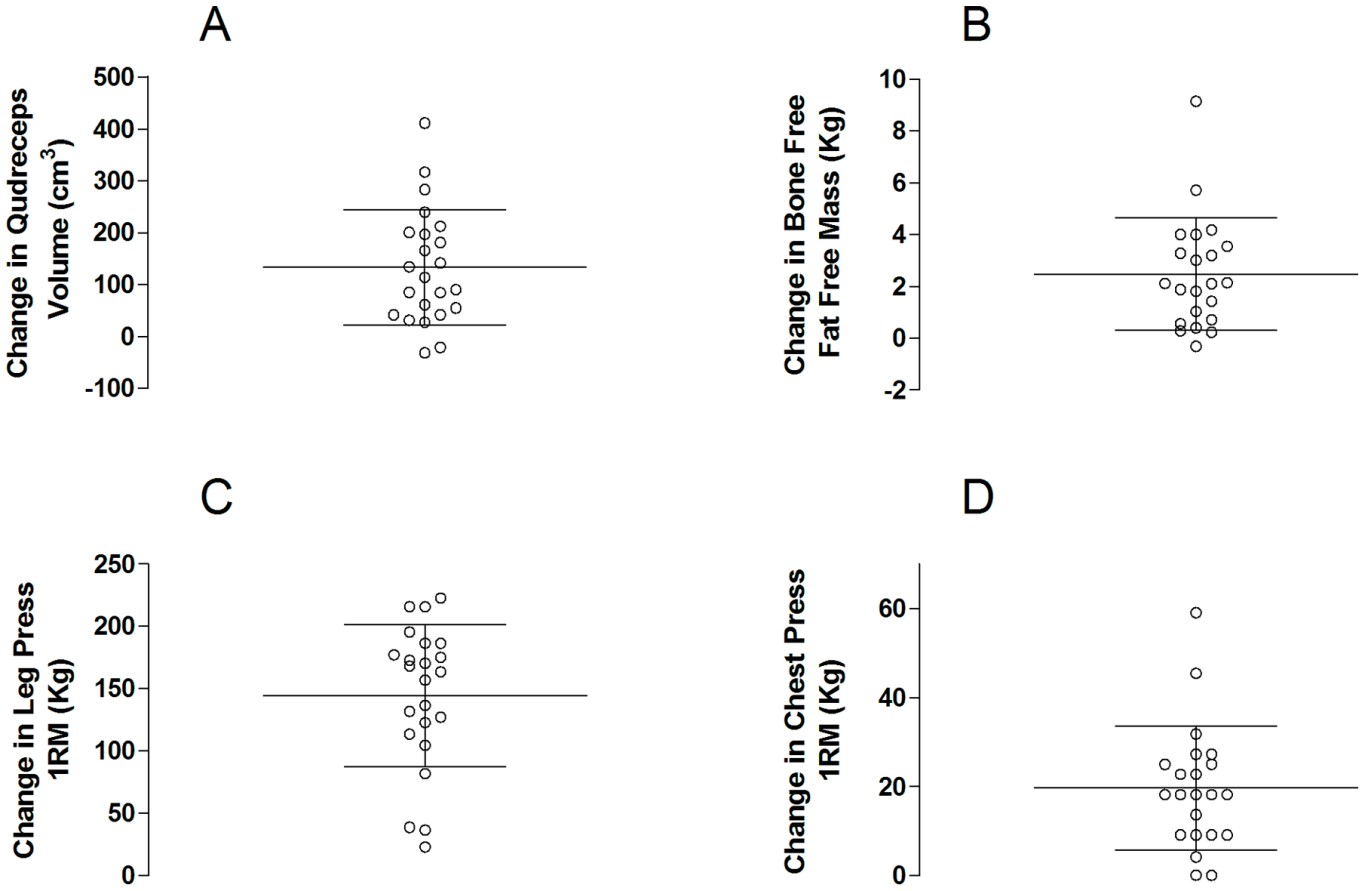
Muscle volume, muscle mass, and strength changes following resistance training. The absolute increase in A) Quadriceps muscle volume determined by MRI, B) Fat free bone free mass determined by DXA, C) Leg press 1RM and D) Chest press 1RM. Each dot represents a single subject, the lines show the group mean change and the standard deviation of the mean. All increases were significantly different from zero (i.e., an increase from pre training P<0.05).

Quadriceps muscle volume increased from 1837±195 to 1970±71 cm^3^ (Figure 1A), while whole body fat- and bone-free mass increased from 62.6±2.0 to 64.8±2.1 kg (Figure 1B). Maximal isotonic strength, expressed as 1 RM, increased from 236±15 to 380±15 kg and from 77±4 to 96±4 kg in the leg press (Figure 1C) and chest press (Figure 1D) exercises respectively.

### Western Blotting

**Figure 2:**
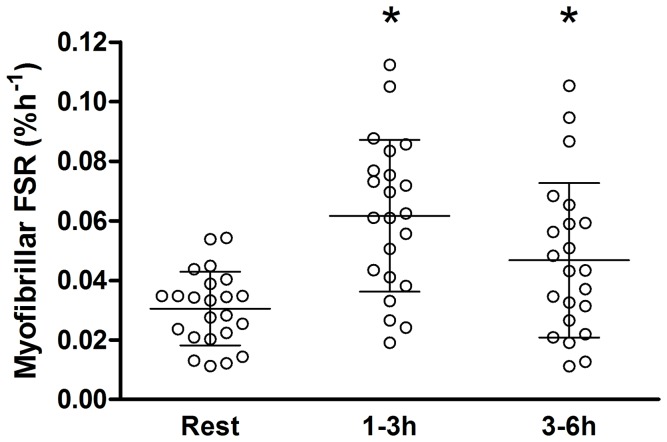
Phosphorylation of anabolic signalizing proteins. The results are expressed as fold changes from rest at 1, 3 and 6) mTOR phosphorylation at Ser2448, B) Akt phosphorylation at Ser473, C) 4E-BP1 phosphorylation at Thr37/46 and D) rpS6 phosphorylation at Ser240/244. * Significantly different from rest P<0.05. † Signficantly different from 1 and 3 hour time points P<0.05.

**Figure 3:**
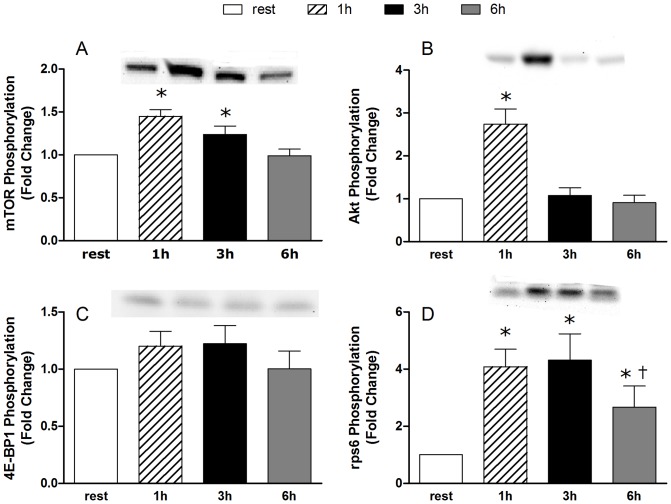
Relationship between muscle hypertrophy and potential correlates. A) The relationship between changes in muscle volume as measured by MRI and the Myofibrillar fractional synthetic rate (FSR) measured from 1 to 6 hours after an acute bout of resistance exercise and nutrition before the start of the resistance training period (r = 0.10, P = 0.67). B) The relationship between changes in muscle volume as measured by MRI and 4E-BP1 phosphorylation at Thr37/46 measured 1 hour after an acute bout of resistance exercise and nutrition before the start of the resistance training period (r = 0.42, P = 0.05).

Phosphorylation of mTOR^Ser2448^ was increased above rest at 1 and 3 h post exercise but had returned to baseline by 6 hours post exercise (Figure 2A). Phosphorylation of Akt^Ser473^ was increased above resting at 1 h post-exercise then returned to baseline by 3 hours post exercise (Figure 2B). Phosphorylation of 4E-BP1^Thr37/46^ was not significantly increased at any time post-exercise (*P* = 0.142; Figure 2C). Phosphorylation of rpS6^Ser240/244^ was elevated above rest at 1,3 and 6 h post-exercise; however, at 6 h post exercise the phosphorylation was reduced compared to 1 and 3 h (Figure 2D). There was a significant correlation between the phosphorylation of 4E-BP1 phosphorylation at 1 h post exercise and the change in muscle volume (r = 0.42, *P* = 0.047, Figure 3B). No correlations were evident between the phosphorylation any of the signalling proteins measured and changes in muscle volume or lean fat-and bone-free mass (data not shown).

### Myofibrillar Protein Synthesis

**Figure 4:**
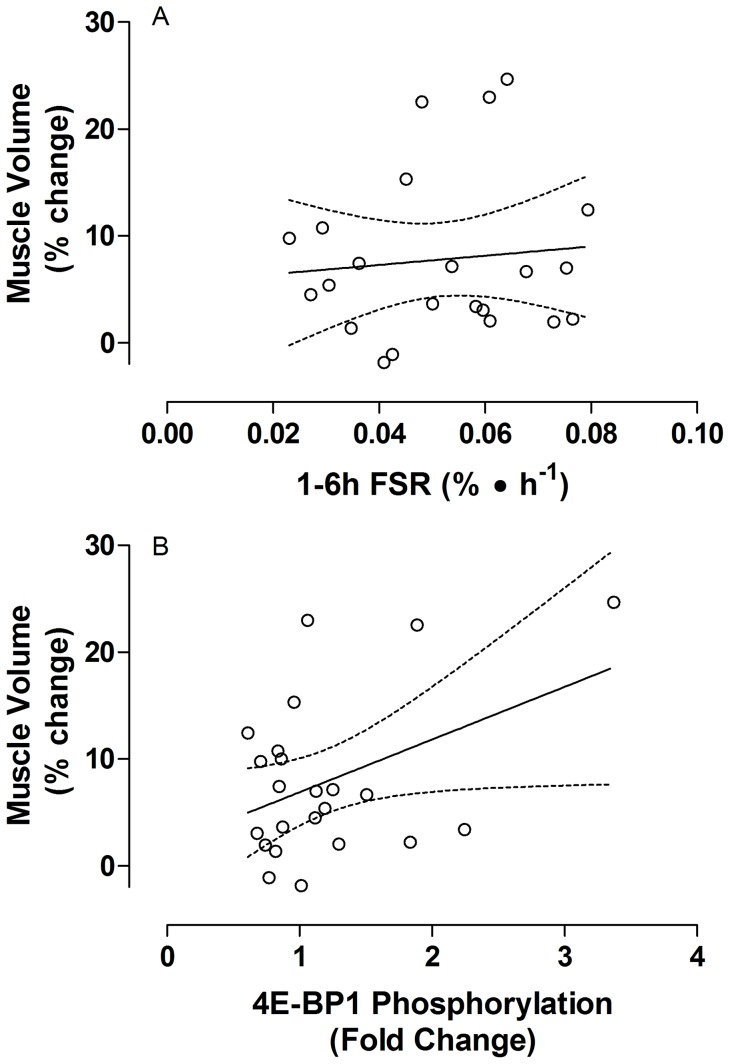
Myofibrillar Protein synthesis. FSR is calculated at rest and after an acute bout of resistance exercise and protein ingestion prior to the start of the resistance training period. The other rates were calculated from 1 to 3–6 hours after the resistance exercises. Each circle, square, and triangle represents a single subject at rest, 1–3 and 3–6 hours post exercises respectively * Significantly different than rest P<0.05.

The rate of MPS following resistance exercise was increased compared to rest and was significantly elevated between 1 and 3 h post exercise (*P*<0.005) and from 3 to 6 h post exercise (*P* = 0.034; Figure 4). There was no statistically significant difference between the 1–3 h and the 3–6 h rates (*P* = 0.159). The aggregate response over the entire post-exercise period (1–6 h) was 0.052±0.04%•h^−1^. There was no correlation between MPS in any of the time periods measured and the change in muscle volume as measured by MRI (data not shown). Figure 3A shows the correlation between MPS measured over the full post exercise infusion period and change in muscle volume (r = 0.01). This comparison is highlighted because it should best reflect the full MPS response after exercise and nutrition. Expressing MPS as a fold change from rest did not results in a correlation with changes in muscle volume over the 1–6 post exercise period (r = −.16, *P>0.05*). In addition, there was not a significant correlation between the change in fat- and bone-free (lean) mass and the aggregate response of myofibrillar protein synthesis measured over 1–6 h post exercise (r = 0.13).

## Discussion

We examined the relationship between the acute fed-state exercise-induced rise in MPS and muscle hypertrophy in the same subject hypothesizing that these variables would be related. Interestingly, we observed no relationship between intra-individual measures of the acute response of MPS following subjects' first exposure to leg resistance exercise and nutrition (which we subsequently had subjects follow throughout their training protocol) and MRI-measured muscle volume or DXA-measured lean body mass. Our finding is actually in agreement with that reported by Mayhew et al. [13] who observed no significant relationship between mixed muscle FSR and hypertrophy in a group of 8 young and 7 older men in which FSR measured 24 h after the first bout of resistance training. It is important for the reader to note that the present study differed from the Mayhew et al. study in both the timing of the measurement and the feeding state of the subjects. In the present study subjects ingested a protein rich beverage immediately after the first and each subsequent training session whereas in the Mayhew et al study subjects were fasted during the FSR measurement and did not consume supplemental protein during training [13]. The protein rich beverage was ingested during training in order to maximize muscle hypertrophy [19] and reduce heterogeneity in the subjects' diet and was consumed during the measurement of acute FSR so the conditions would be as similar as possible to the training. Although it was not possible to fully control the subjects' diets during the training period previous work has shown that subjects in this type of study consume adequate protein and calories and variation in diet does not explain variation in post training hypertrophy [20]. Diet records collected on a subset of subjects in this study support the previous finding. Similarly differences in habitual activities outside of the study workouts could have impacted hypertrophy however, subjects participated in a maximum of two pre week of sporting activity outside of the study so it is unlikely this had a major impact.

In previous work, acute responses of MPS to differing nutrition [4], [6], contraction intensity [2], [7], and contraction volume [2], [7] were found to align with chronic training-mediated changes in hypertrophy in studies employing roughly equivalent nutritional and/or contractile conditions preformed but in different sets of subjects. The absence of a significant correlation between the acute early measure of MPS in the untrained state and chronic hypertrophy, in the present study, could be explained by a number of subject-specific changes in the MPS response in terms of: magnitude at times later during the training program, specificity of the protein fraction-specific (i.e., myofibrillar vs. non-myofibrillar MPS response), and/or duration of the response of MPS during the course of training, variations in net muscle protein balance due to differential responses in muscle proteolysis. Clearly, however, acute early measures of MPS are not proxy measures for hypertrophy or hypertrophic potential within the same individual.

Cross-sectional comparisons of trained with untrained persons show that increases in mixed muscle protein FSR were smaller in magnitude, as were increases in mixed muscle proteolysis, in response to resistance exercise [21]. Tang et al. showed that the mixed muscle protein FSR to a bout of resistance exercise with feeding, performed at the same relative intensity pre- and post-training, produced a slightly higher FSR immediately post exercise (90–270 min post exercise) in the trained state, however, the duration of the response was reduced [22]. In contrast, exercise at the same relative intensity resulted, when combined with feeding, results in a lower mixed muscle protein FSR after training when the same absolute intensity was utilized [23]. When examining myofibrillar as opposed to mixed muscle FSR, there were no differences in acute MPS between the trained and untrained conditions after exercise in the fasted state [24]. Taken together, resistance training appears to reduce the duration, but not the amplitude, of the myofibrillar protein synthetic response. Nonetheless, one conspicuous adaptation with resistance training is a ‘refining’ of the synthetic response to emphasize synthesis of myofibrillar proteins and reductions in both sarcoplasmic and mitochondrial protein synthesis in response to contraction [24], [25]. Further research to delineate if responses of MPS play a role in determining hypertrophy would obviously have to include a more protracted time course of the MPS response and look at whether those gaining more lean mass were able to sustain a greater duration of their MPS response during the RT period. It is possible that high and low responders to RT may have similar acute FSR responses to the first bout of exercise but may significantly diverge in terms of FSR response at a point in the training period. A difference in muscle satellite cell content and the degree of myonuclear addition have been shown to relate to the magnitude of RT-induced hypertrophy [9]. It is conceivable that the individual variation in the change in FSR throughout the training period could be related to the degree of myonuclear addition.

A possibility is that subjects gaining more muscle mass with resistance training had a greater suppression of proteolysis as it is net muscle protein balance (i.e., MPS minus MPB) that would, strictly speaking, determine gains in muscle mass. Work by Glynn and associates shows that there are increases in MPS and reductions MPB in response to a combination of feeding and resistance exercise, however, the magnitude of the changes in MPS is ∼4–5 fold greater than the change in MPB [26]. Similar differences in magnitude of the response of MPS relative to MPB have been seen with resistance exercise alone [27]. These data suggest that changes in MPS is the main locus of control and is far more responsive to nutritional and contractile stimuli in regulating, changes in muscle size than MPB. In addition, when measures of mixed muscle protein synthesis and breakdown have been made in the post-exercise period in the same subjects a reasonably good correlation exists between the two variables [21], [27] which does not point to a measurable divergence in regulation but rather a link between the two processes. However, we cannot rule out the possibility that changes in protein breakdown could regulate gains in lean mass with resistance training.

The Akt-mTORC-1 pathway is an important regulatory pathway for muscle hypertrophy and is considered necessary for protein synthesis [28]. Phosphorylation of proteins within this pathway such as P70S6K, rpS6 and 4E-BP1 may show stronger relationships with hypertrophy because they are downstream from mTOR in the signalling pathway that culminates in protein translation [13]. There are multiple reports of correlations between P70S6K and hypertrophy in the literature, however, these correlations tend to be weak [12], [13] or have a small sample size [1]. Our lab has shown that significant muscle hypertrophy is possible with RT even when there is phosphorylation of P70S6K one hour after the first exercise bout [2], we have also shown that in one study P70S6K phosphorylation six hours after the first exercise bout is weakly related to training mediated hypertrophy [12]. These findings indicate how fickle a single time point measurement can be and provide a justification for our initial hypothesis that dynamic measurement such as MPS would show a greater relationship with hypertrophy.

Previous work from our lab has shown a correlation between acute phosphorylation of 4E-BP1 and myofibrillar FSR after resistance exercise [7]. In the current study we did not, however, see a relationship between FSR and 4E-BP1 phosphorylation but did see a relationship between hypertrophy and 4E-BP1 phosphorylation. Eukaryotic initiation factor 2B epsilon is another member of the Akt-mTOR pathway and has been proposed to be a regulator or variability in human hypertrophy [29]. However, technical limitations did not allow for the measurement of acute phosphorylation status of this target and the small amount of human data available does not suggest a linear relationship between hypertrophy and changes in protein abundance [29]. In a large study with young and old subjects the phosphorylation of multiple proteins in the Akt-mTOR pathway were shown to be unrelated to resistance exercise-induced changes in lean body mass [3]. Because anabolic signaling is transient and measured at discreet time points, and the degree of phosphorylation may not reflect activity it is doubtful that phosphorylation of a single protein could explain a large proportion of the variance in muscle hypertrophy [3].

The results from this study indicate that acute measurements of MPS over 6 hours following exercise and nutrition are not predictive of muscle hypertrophy following 16 weeks of resistance training and supplement ingestion in the same subjects. It is possible that the measures of MPS at later time-points following acute exercise may demonstrate a correlative relationship with muscle hypertrophy. However, the magnitude and duration on the MPS response measured within the same individual appears to change with training [22], [24]. It is possible the some subjects may maintain a robust MPS response throughout the training period whereas some subjects may show a diminished MPS response after training. Because data from the present study does not show a relationship between acute measures of MPS and skeletal muscle hypertrophy, it is likely that changes in MPS with training are not uniform between subjects. A systems biology approach incorporating proteomics, genomic, or transcriptomics may be required prospectively to estimate hypertrophy or hypertrophic potential.
