# Correction: Acute Post-Exercise Myofibrillar Protein Synthesis Is Not Correlated with Resistance Training-Induced Muscle Hypertrophy in Young Men

**DOI:** 10.1371/journal.pone.0098731

**Published:** 2014-05-21

**Authors:** The PLOS ONE Staff

Figures 2, 3, and 4 are switched. Figure 3 should be Figure 2, Figure 4 should be Figure 3 and Figure 2 should be Figure 4. The authors have provided corrected versions and the relevant legends here.

**Figure 2:**
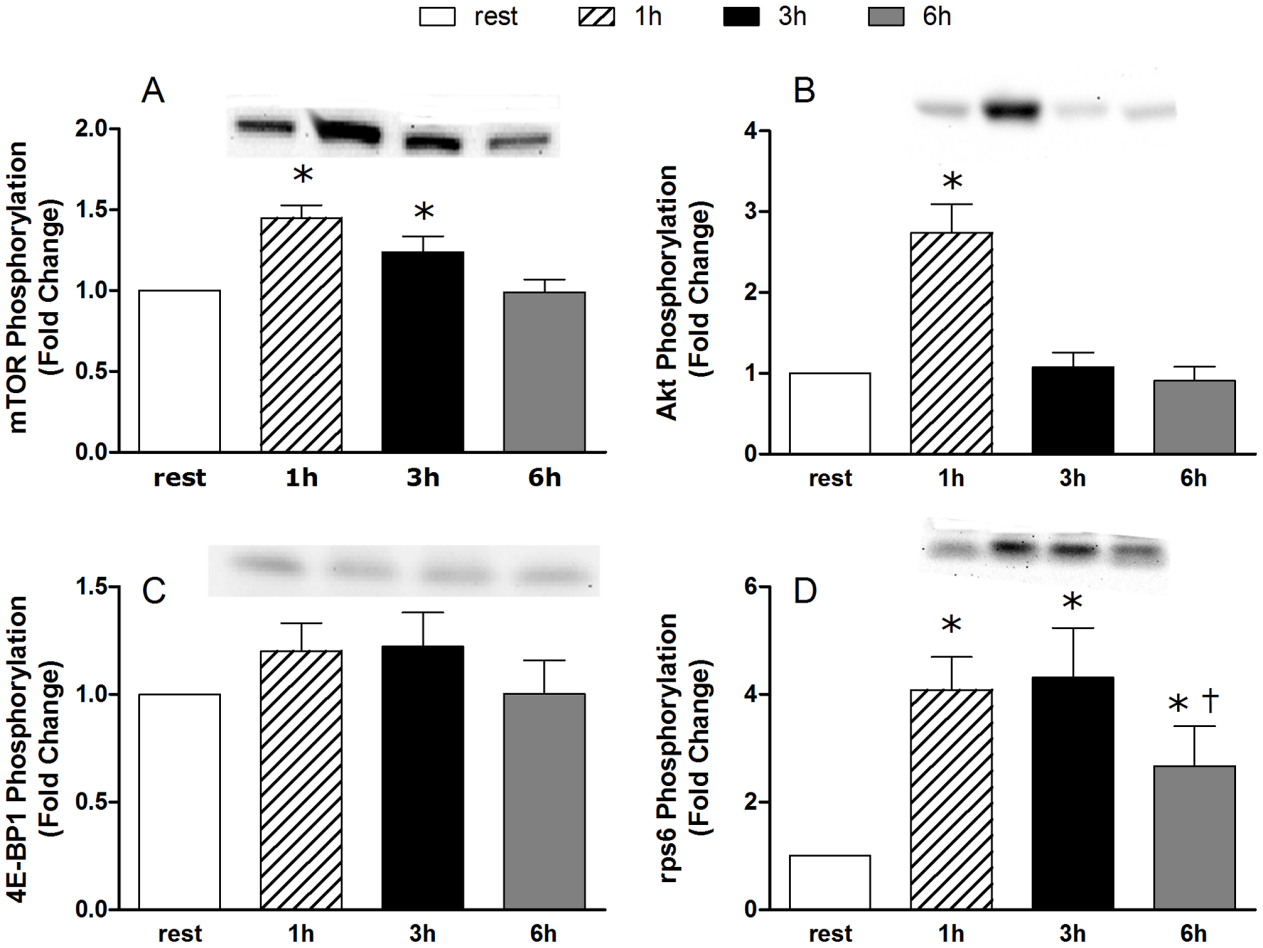
Phosphorylation of anabolic signalizing proteins. The results are expressed as fold changes from rest at 1, 3 and 6) mTOR phosphorylation at Ser2448, B) Akt phosphorylation at Ser473, C) 4E-BP1 phosphorylation at Thr37/46 and D) rpS6 phosphorylation at Ser240/244. * Significantly different from rest P<0.05. † Signficantly different from 1 and 3 hour time points P<0.05.

**Figure 3:**
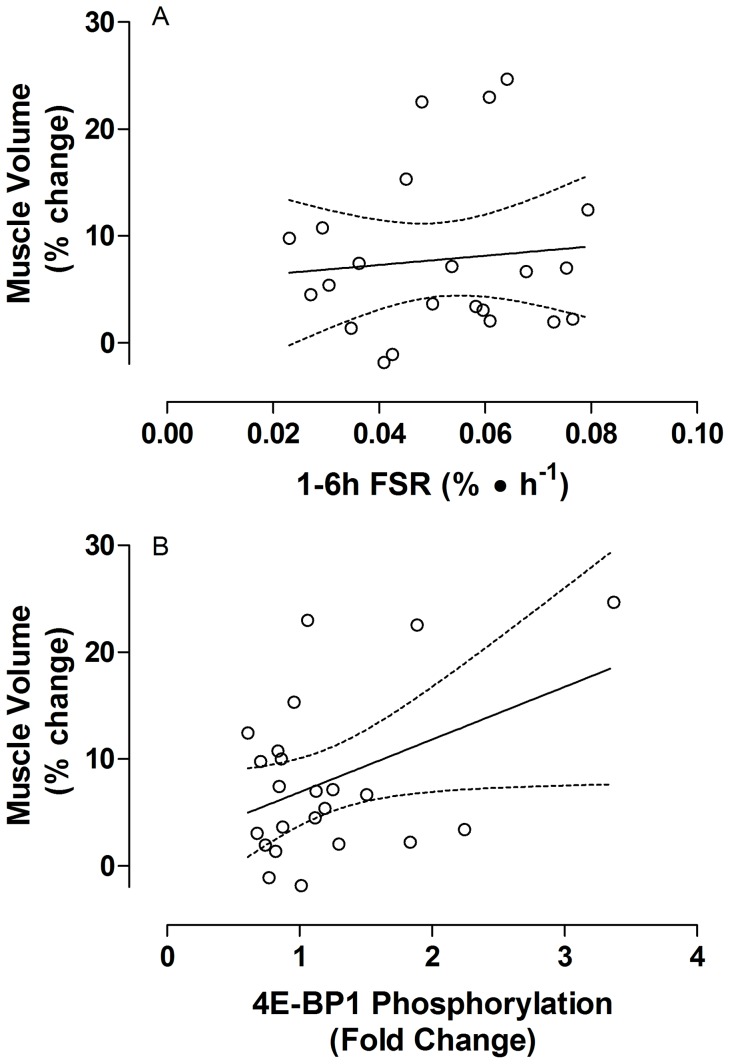
Relationship between muscle hypertrophy and potential correlates. A) The relationship between changes in muscle volume as measured by MRI and the Myofibrillar fractional synthetic rate (FSR) measured from 1 to 6 hours after an acute bout of resistance exercise and nutrition before the start of the resistance training period (r  =  0.10, P  =  0.67). B) The relationship between changes in muscle volume as measured by MRI and 4E-BP1 phosphorylation at Thr37/46 measured 1 hour after an acute bout of resistance exercise and nutrition before the start of the resistance training period (r  =  0.42, P  =  0.05).

**Figure 4:**
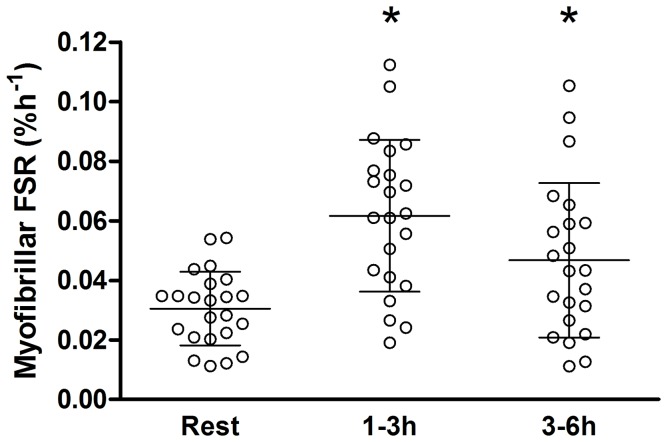
Myofibrillar Protein synthesis. FSR is calculated at rest and after an acute bout of resistance exercise and protein ingestion prior to the start of the resistance training period. The other rates were calculated from 1 to 3–6 hours after the resistance exercises. Each circle, square, and triangle represents a single subject at rest, 1–3 and 3–6 hours post exercises respectively * Significantly different than rest P<0.05.
